# Treatment of ureteropelvic junction obstruction in patients with renal calculi via laparoscopic pyeloplasty and flexible vacuum-assisted ureteral access sheath ureteroscopy: a multicenter retrospective observational study

**DOI:** 10.1186/s12894-024-01453-4

**Published:** 2024-03-26

**Authors:** Yang Mi, Zhiqin Kang, Jingyu Wang, Liang Yan, Jun Zhang

**Affiliations:** 1 Department of Urology, Shanxi Academy of Medical Sciences, Shanxi Bethune Hospital, Taiyuan, Shanxi People’s Republic of China; 2 Department of Emergency, Shanxi Academy of Medical Sciences, Shanxi Bethune Hospital, Taiyuan, Shanxi People’s Republic of China; 3 First hospital of shanxi medical university, No. 85 Jiefangnan Road, Taiyuan, 0300001 Shanxi People’s Republic of China

**Keywords:** Ureteropelvic junction obstruction, Laparoscopic pyeloplasty, Flexible ureteroscopy, Flexible vacuum-assisted ureteral access sheath

## Abstract

**Background:**

Ureteropelvic junction obstruction (UPJO) is a common obstructive disease of the urinary tract. UPJO patients commonly exhibit coexistent renal calculi. The main aim of therapy is to relieve the obstruction and remove the stones at the same time.

**Methods:**

This retrospective study included 110 patients diagnosed with UPJO coexisting with multiple renal calculi at Shanxi Bethune Hospital and the First Hospital of Shanxi Medical University between March 2016 and January 2022. Patients were divided according to the methods used for dealing with UPJO and renal calculi. In Group A, patients underwent traditional open pyeloplasty and pyelolithotomy. In Group B, patients underwent percutaneous nephrolithotomy first and then laparoscopic pyeloplasty. In Group C, patients underwent flexible cystoscopy to remove stones and then laparoscopic pyeloplasty. In Group D, patients underwent flexible vacuum-assisted ureteral access sheath (FV-UAS)assisted flexible ureteroscopy (f-URS) and underwent laparoscopic pyeloplasty. The stones were broken up using a holmium laser. The pyeloplasty success rate, stone clearance rate, operation time, bleeding amount, complication occurrence rate, postsurgical pain, length of stay, and hospitalization cost were compared between the groups. The follow-up period was at least 2 years.

**Results:**

The use of f-URS and the FV-UAS, significantly increased the renal stone clearance rate and significantly reduced the complication incidence and operation time in UPJO patients with multiple coexisting renal calculi.

**Conclusions:**

Laparoscopic pyeloplasty combined with f-URS and FV-UAS is safe and effective for treating UPJO in patients complicated by renal caliceal stones.

**Trial registration:**

Retrospectively registered.

## Background

Ureteropelvic junction obstruction (UPJO) is a common obstructive disease of the urinary tract. It is very common for UPJO patients to have coexistent renal calculi [1]. The main aim of therapy is to relieve the obstruction and remove the stones at the same time. Open pyeloplasty and pyelolithotomy have been the standard treatments for this disease in recent decades [2]. Currently, with the development of minimally invasive urology, laparoscopic pyeloplasty (LP) and robot-assisted laparoscopic pyeloplasty (RAP) are the most commonly performed surgical intervention for reliving stenosis [3, 4]. However, compared to traditional pyelolithotomy, an efficient and safe method for removing renal calculi, especially multiple renal stones, is still needed. Previous studies have shown that percutaneous nephrolithotomy and flexible cystoscopy may efficiently solve this problem [4, 5]. In this study, we demonstrated the clinical efficacy of using LP and a novel flexible vacuum-assisted ureteral access sheath (FV-UAS) during single-use flexible ureteroscopy(f-URS) in treating UPJO patients with coexisting multiple renal stones.

## Methods

This retrospective study included 110 patients diagnosed with UPJO and multiple coexisting renal calculi at Shanxi Bethune Hospital and the First Hospital of Shanxi Medical University between March 2016 and January 2022. The patients were diagnosed by intravenous pyelography (IVP) and computed tomography (CT). The diagnostic criteria for ureteral obstruction were a half-time of more than 20 min after diuretic therapy on a renal scan and delayed nephrogram or excretion with hydronephrosis on radiological examination [6]. All the patients’ renal stones were measured accurately via the automated 3D volume calculations based on computerized tomography performed by Thomas Tailly [7] and were broken up using a holmium laser.

All patients underwent surgery under general anaesthesia. In Group A, patients underwent traditional open pyeloplasty and pyelolithotomy. A 7.0 F double-J tube remained in place for 8 weeks, and a drain was placed in the retroperitoneal space. To ensure low pressure in the renal pelvis, the catheter remained in place for 7 days. The catheter and drain were removed in sequential order when the drain output was less than 20 ml for 24 h.

The UPJO in Groups B, C, and D were treated with LP. Patients were placed in the lateral decubitus position with the affected side facing upwards, and pneumoperitoneum was established via a Veress needle positioned in the umbilicus. The pneumoperitoneum pressure was maintained at 14 mm Hg by carbon dioxide, and when the pressure was stable, a classic three-port transperitoneal laparoscopy was performed. A 12-mm trocar for the camera was placed 30 mm superior to the umbilicus and lateral to the rectus muscle, and the two main operation trocars (5 and 12 mm) were placed 20 mm inferior to the costal margin of the left collarbone midline and 30 mm interior and superior to the anterior superior iliac spine. After the renal calculi were removed, the redundant pelvis tissue was cut using the method described by Kunlin Yang et al. [8], and standard Anderson–Hynes pyeloplasty was performed using a 4–0 Vicryl suture. The indwelling double-J tube, catheter and drain placement times were the same as Group A.

In Group B, patients underwent percutaneous nephrolithotomy (PCNL) to remove renal stones before LP. PCNL was performed with patients in a standard prone position, and puncture was performed as described by Thomas Knoll in a previous study [9]. Most patients underwent single-tract PCNL, and multiple accesses were also accepted for maximum clearance of the calculi.

**Fig. 1:**
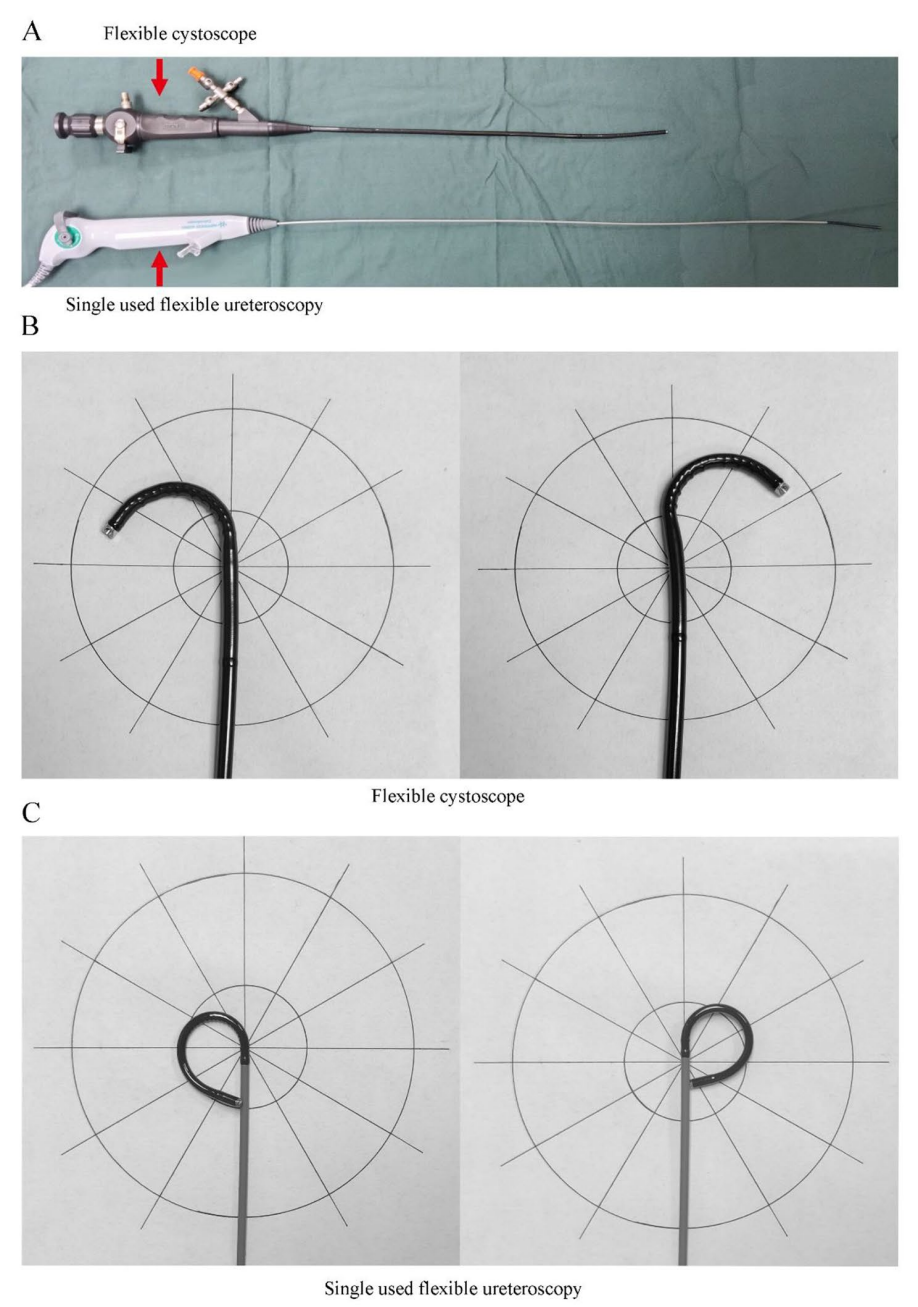
**A**. Total length of flexible cystoscope and single use flexible ureteroscopy; **B**. Maximal deflection of flexible cystoscope; **C**. Maximal deflection of single use flexible ureteroscopy

In Group C, patients underwent surgery via a mini-incision in the renal pelvis under the laparoscope, and the flexible cystoscope was inserted into the pelvis to extract the pelvic and calyceal stones through a 12-mm trocar. The mini-incision was not suitable for the final pyeloplasty, but it helped decrease the extravasation of the flushing agent when the cystoscope was used (shown in Fig. 1). When the clearance of stones was confirmed by cystoscopy, the patients underwent pyeloplasty.

**Fig. 2:**
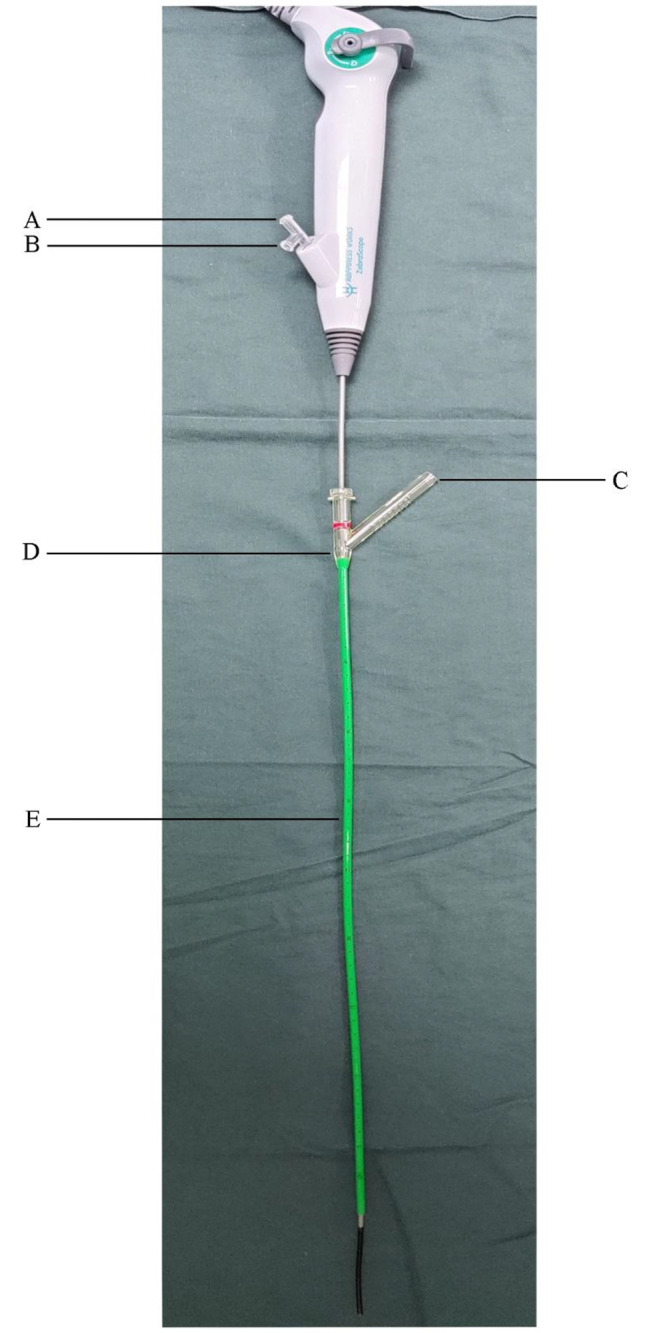
The single used flexible ureteroscopy and suction ureteral access sheath. **A**. Work channel of single used flexible ureteroscopy; **B**. The irrigation fluid irrigation channel; **C**. Channel for vacuum suction; **D**. Tail of the suction ureteral access sheath; **E**. The scale on the surface of the suction ureteral access sheath, it could indicate the placement depth in uretero

Patients in Group D underwent surgery via a mini-incision in the renal pelvis under the laparoscope, and the vacuum suction urethral access sheath (11 [inner diameter]/13 [external diameter] Fr; shown in Fig. 2) was inserted into the renal pelvis through a 12-mm trocar. Under direct vision of the single-use f-URS (shown in Fig. 1), the pelvic and calyceal stones were broken up using a holmium laser. The pieces of the stones were removed by the FV-UAS. Any stone pieces larger than 4 mm were extracted using a nitinol basket. Upon confirmation of complete stone clearance, the patients underwent pyeloplasty.

All patients were required to be followed up every 3–6 months after the operation in the first year and once a year thereafter. In the first year after the operation, patients underwent renal ultrasonography every 3 months and IVP every 6 months after the operation. Beginning in the second year after the operation, patients underwent IVP once a year. The postoperative outcome was evaluated by IVP mainly because the cost of IVP was significantly lower than that of CT. The follow-up period was at least 2 years. IVP and renal ultrasonography were necessary if patients had uncomfortable symptoms or pain in the flank. Surgery was considered successful if there was no residual renal stone larger than 4 mm in diameter, if pelvic/calyceal dilatation decreased on IVP (as determined by measuring the size of the pelvis/calyceal), or if renal drainage/function improved renographically (a radioisotope outflow level of the maximum activity within the first 30 min after radionuclide application was measured) [10, 11].

Patient characteristics, complication rate, bleeding amount, stone clearance rate, hospitalization cost, postsurgical pain (evaluated 1 week later after the operation, measured using a visual analogue scale, VAS) and pyeloplasty success rate were compared among all groups. Differences in the measured data were compared using a single-way analysis of variance after normality testing, the qualitative data were analyzed using the chi-square test, and the ranked data were analyzed by the rank-sum test. Differences were considered statistically significant if the *P* value was < 0.05.

## Results

### Patients characteristics

There was no significant difference among the groups in terms of mean age (A, 23.21 ± 4.11 years; B, 28.53 ± 9.14 years; C, 26.19 ± 8.86 years; D, 25.19 ± 11.81 years); sex ratio (female/male, A, 13/12; B, 11/10; C, 16/17; D, 17/14); body mass index (A,25.53 ± 1.82 kg/m^2^; B, 23.18 ± 3.61 kg/m^2^; C, 26.55 ± 3.34 kg/m^2^; D, 25.08 ± 3.86 kg/m^2^) ;total stone volume (A, 3.42 ± 0.16 cm^3^; B, 3.96 ± 0.21 cm^3^; C, 4.11 ± 0.51 cm^3^; D, 3.89 ± 0.41 cm^3^); stone volume in the renal pelvis; or stone volume in the upper, middle, or lower renal calyx. There was one 13 year-old patient in Group D. The preoperative clinical and demographic profiles of the patients are shown in Table 1.

**Table 1:** Patients’ preoperative clinical and demographic details

	Group A (*n* = 25)	Group B (*n* = 21)	Group C (*n* = 33)	Group D (*n* = 31)
Mean age ± SD (year)	23.21 ± 4.11^abc^	28.53 ± 9.14^ab^	26.19 ± 8.86^a^	25.19 ± 11.81
Female/male	13/12^abc^	11/10^ab^	16/17^a^	17/14
Body mass index (BMI) ± SD	25.53 ± 1.82^abc^	23.18 ± 3.61^ab^	26.55 ± 3.34 ^a^	25.08 ± 3.86
Total stone volume (cm^3^) ± SD	3.42 ± 0.16^abc^	3.96 ± 0.21^ab^	4.11 ± 0.51 ^a^	3.89 ± 0.41
Stone volume in renal pelvis (cm^3^) ± SD	1.86 ± 0.13^abc^	1.92 ± 0.09^ab^	1.83 ± 0.16 ^a^	1.92 ± 0.21
Stone volume in upper renal calyx (cm^3^) ± SD	0.51 ± 0.09^abc^	0.63 ± 0.11^ab^	0.74 ± 0.06 ^a^	0.66 ± 0.08
Stone volume in middle renal calyx (cm^3^) ± SD	1.11 ± 0.12^abc^	1.36 ± 0.11^ab^	1.41 ± 0.31 ^a^	1.39 ± 0.26
Stone volume in lower renal calyx (cm^3^) ± SD	1.58 ± 0.21^abc^	1.96 ± 0.43^ab^	1.81 ± 0.36 ^a^	1.69 ± 0.33

Abbreviations: a, compared to group D, *P* > 0.05; b, compared to group C, *P* > 0.05; c, compared to group B, *P* > 0.05

### Treatment efficacy

Successful surgery was considered successful if pyeloplasty was successful and the patient was stone free. The surgery success rates in Groups A, B, C and D were 68.00, 85.71, 78.79 and 94.29%, respectively. The difference in stone clearance rates among the four groups was significant. The stone clearance rate and pyeloplasty success rate were also used to assess treatment efficacy. The stone clearance rates in Groups A, B, C and D were 68.00, 85.71, 78.79 and 94.29%, respectively. The difference in stone clearance rates among the four groups was significant. The pyeloplasty success rates in Groups A, B, C and D were 92.00, 95.24, 93.94, and 94.29%, respectively. The pyeloplasty success rates were not significantly different among the four groups. The clinical profiles of the patients are shown in Table 2.

**Table 2:** The stones clearance rate and success rate of pyeloplasty

	Group A (*n* = 25)	Group B (*n* = 21)	Group C (*n* = 33)	Group D (*n* = 31)
Stone clearance rate	17/25(68.00%)^abc^	18/21(85.71%)^ab^	26/33(78.79%)^a^	33/35(94.29%)
Success rate of pyeloplasty	23/25(92.00%)^def^	20/21(95.24%)^de^	31/33(93.94%)^d^	33/35(94.29%)
Success rate of operation	17/25(68.00%)^abc^	18/21(85.71%)^ab^	26/33(78.79%)^a^	33/35(94.29%)

Abbreviation: a, compared to group D, *P* < 0.05; b, compared to group C, *P* < 0.05; c, compared to group B, *P* < 0.05; d, compared to group D, *P* > 0.05; e, compared to group C, *P* > 0.05; f, compared to group B, *P* > 0.05

### Patients postoperative clinical details

The operation time was ranked fist for Group B (189.41 ± 25.62 min), second for Group C (153.75 ± 26.13 min), third for Group D (138.85 ± 26.13 min) and fourth for group A (189.41 ± 25.62 min). The differences among the four groups were significant.

The amount of bleeding was 52.31 ± 5.81 ml in Group A, 89.84 ± 15.6 ml in Group B, 23.45 ± 13.33 ml in Group C and 25.12 ± 12.87 ml in Group D. Compared to that in Group D, the amount of bleeding was not significantly different in Group C but was significantly greater in Groups A and B. Compared with that in Group A, the amount of bleeding in Group B was significantly greater.

The operation-related complications mainly included perirenal infection, urinary extravasation, and acute renal injury. In Group A, three patients suffered operation-related complications (3/25, 12.00%), two patients suffered both perirenal infection and urinary extravasation and one patient suffered a perirenal infection, urinary extravasation and acute renal injury at the same time. In Group B, four patients suffered operation-related complications (4/21, 19.05%), one patient suffered both a perirenal infection and urinary extravasation and three patients suffered a perirenal infection, urinary extravasation and acute renal injury at the same time. In Group C, three patients suffered operation-related complications (3/33, 9.09%), two patients suffered both perirenal infection and urinary extravasation and one patient suffered an acute renal injury. In Group D, two patients suffered operation-related complications (2/31, 6.45%), one suffered both a perirenal infection and urinary extravasation, and one suffered acute renal injury. The order from the highest to lowest operation-related complication occurrence rate was Group B, Group A, Group C and Group D. The differences among the groups were significant.

Postoperative pain was evaluated 1 week after surgery using the VAS. The VAS score was 6.61 ± 2.19 in Group A, 4.93 ± 1.85 in Group B, 2.92 ± 1.93 in Group C and 3.01 ± 1.63 in Group D. Compared with that in group D, the VAS score was not significantly different in Group C but was significantly higher in Groups A and B. Compared with that in group B, the score in Group A was significantly higher.

The LOS was 14.09 ± 3.67 days in Group A, 16.13 ± 4.23 days in Group B, 11.89 ± 3.91 days in Group C, and 10.99 ± 4.67 days in Group D. Compared with that in Group D, the LOS was not significantly different in Group C but was significantly longer in Groups A and B. Compared with that in group B, the LOS in Group A significantly longer.

The hospitalization costs were $2669.84 ± 342.89 in Group A, $3811.20 ± 433.82 in Group B, $3289.56 ± 564.23 in Group C and $4156.14 ± 459.58 in Group D. The difference in operation time among the four groups was significant.

The follow-up durations were not significantly different among the four groups. The clinical profiles of the patients are shown in Table 3.

**Table 3:** Patients’ postoperative clinical and demographic details

	Group A (*n* = 25)	Group B (*n* = 21)	Group C (*n* = 33)	Group D (*n* = 31)
Operation time (minute)	126.44 ± 32.51^abc^	189.41 ± 25.62^ab^	153.75 ± 26.13^a^	138.85 ± 26.13
Bleeding amount (ml)	52.31 ± 5.81^abc^	89.84 ± 15.61^ab^	23.45 ± 13.33	25.12 ± 12.87
Complication rate	3/25(12.00%)^abc^	4/21(19.05%)^ab^	3/33(9.09%)^a^	2/31(6.45%)
(1) Perirenal infection and extravasation	3	4	2	1
(2) Acute renal injury	1	3	1	1
Post-surgical pain (Visual Analogue Scale, VAS)	6.61 ± 2.19^abc^	4.93 ± 1.85^ab^	2.92 ± 1.93	3.01 ± 1.63^ef^
Length of stay (LOS, day)	14.09 ± 3.67^abc^	16.13 ± 4.23^ab^	11.89 ± 3.91	10.99 ± 4.67
Hospitalization cost (dollar)	2669.84 ± 342.89^abc^	3811.20 ± 433.82^ab^	3289.56 ± 564.23^a^	4156.14 ± 459.58
Follow-up duration (month)	29.19 ± 8.21^def^	32.03 ± 10.19^de^	28.68 ± 11.13^d^	31.31 ± 6.67

Abbreviation: a, compared to group D, *P* < 0.05; b, compared to group C, *P* < 0.05; c, compared to group B, *P* < 0.05; d, compared to group D, *P* > 0.05; e, compared to group C, *P* > 0.05; f, compared to group B, *P* > 0.05

## Discussion

UPJO is one of the most common urogenital congenital obstructive diseases. It has been reported that approximately 16–30% of UPJO patients have coexisting renal calculi^[1]^. The effective relief of UPJO and removal of renal stones are the keys to successful treatment. Open pyeloplasty and pyelolithotomy are considered standard treatments in most medical centres. As minimally invasive urologic technologies advance, LP, RAP and antegrade balloon dilation have been demonstrated to be effective in relieving stenosis [12]. LP has been widely used in recent years because of its curative effect on exact and minimum injuries; some researchers even consider LP to be the “gold standard” for managing UPJO [13]. However, there is no consensus regarding the most efficient method for resolving renal calculi in UPJO patients.

In this research, the pyeloplasty success rates were not significantly different among the groups. The results revealed that LP and open pyeloplasty had the same effect on reliving the obstruction. We found that UPJO patients always had multiple coexisting renal calculi, and the size of was stones less than 2 cm. These stone features led us to believe that the stones they were easily distributed in the calyces.

The stone clearance rate was the lowest in the open surgery group because the stones in the calyces were easily missed as it was difficult to explore the calyces through the pelvic incision. On the other hand, the operation time and hospitalization cost were the lowest. Because the operative field in open surgery may be wider than that in other surgical methods and because assistants could help the surgeons finish some work to reduce the operation time, the efficacy of surgery improved. Moreover, open surgery requires the use of endoscopic equipment, so the hospitalization cost significantly decreases. However, the postsurgical pain score was highest in the open surgery group, as the incision was the largest. We may consider that traditional surgery is more suitable for treating UPJO patients with coexisting renal stones, especially those suffering financial hardship.

Percutaneous nephrolithotomy improved the stone clearance rate compared to that in Groups A and C, but was just lower than that in Group D. Using this method, multiple puncture panels may be necessary because it is difficult to access multiple renal calyces stones. However, multiple puncture panels significantly increase the risks of bleeding and acute renal injury. The operation time in Group B was the longest because patients first underwent PCNL in the prone position and were placed in the lateral decubitus position to undergo LP. As the puncture was guided by ultrasound, not direct vision, it may lead to injuries to the renal sinus arteries and veins. The high rates of perirenal infection and extravasation may be related to intraoperative renal irrigation, as the irrigation solution extravasated through the puncture panel. The LOS was the longest in Group B because patients who underwent PCNL were on bedrest for 5–7 days in case of surgery-related delayed renal haemorrhage. Therefore, as the operation time, bleeding amount, complication rate, and LOS were the highest among the four groups, PCNL may be more suitable for patients with good general physical health, especially those with single renal calyx stones or multiple pelvic stones.

Many researchers have reported the efficiency of renal stone clearance using a flexible cystoscope in UPJO patients, and we experienced the same outcome in this research. Compared with that in Group A, the traditional open surgery group, the stone clearance rate in Group C was significantly higher but lower than that in groups B and D. In addition, we also found that the bleeding volume, complication rate, VAS score, and LOS were significantly lower in Group C than in Groups A and B, demonstrating that the flexible cystoscope was a less invasive operation method. However, there is still a deficiency of this technique, as the use of flexible guiding tubes increase the operation time, the complexity of the procedure and the difficulty of controlling the flexible cystoscope [14]. In addition, plenty of washing fluid is needed and timely clearance of washing fluid is necessary to decrease the incidence of extravasation related perirenal infection.

As the results showed, compared to those of flexible cystoscopy, the operation times and complication rates of FV-RAS and f-RUS were significantly shorter and lower, and both the stone clearance rate and the hospitalization cost higher. The f-RUS was better for dealing with multiple calyceal calculi, especially in patients whose calculi were located at the acute infundibulo-ureteropelvic angle (IUPA) or the narrow infundibular neck of the calyx. The f-RUS had a larger active tip deflection that could reach 270° up or down and a slender size (tip/shaft, 7.2/8.5 F). The FV-RAS was placed into the pelvis through the small incision made near the location targeted stones removals. The urethral access sheath helps control the f-RUS, and vacuum suction significantly decrease the risk of extravasation of washing fluid during the operation. Moreover, tiny fragments of stones can be sucked out directly through the sheath, which significantly decreases the time needed for extracting stones using a nitinol basket. Therefore there was a direct correlation between the highest surgery success rate and the highest stone clearance rate in Group D. However, the increase in hospitalization costs associated with single-use of the f-RUS (1050 dollars) and FV-RAS (150 dollars) may restrict further application of these systems.

The traditional open surgery procedure has significantly lower costs, but the renal stone clearance rates were low, and the surgical scars were significant. The renal stone clearance rate and the cost of PCNL seems acceptable, but the high complication rate is still a concern that cannot be ignored for most patients. The curative effect of LP and FV-RAS assisted f-RUS seemed to be the most favourable. However, most Chinese patients cannot afford treatment. All patients were fully informed of the advantages and disadvantages of each therapy before the operation. The final therapy was decided according to the medical diagnosis and with full respect for the wishes of the patients.

## Conclusions

This study compared different methods of treating UPJO patients with coexisting renal calculi and demonstrated the safety and efficacy of using LP and a novel FV-RASduring single-use f-RUS. This method decreases the infection risk and may help achieve better renal stone clearance rates. This study is limited by its retrospective design and the small number of patients. However, further clinical observation of a larger population of patients is needed to determine the safety and efficacy of the treatment.

## Data Availability

The data that support the findings of this study are available from the corresponding author upon reasonable request.

## Abbreviations

UPJO: Ureteropelvic junction obstruction
LP: Laparoscopic pyeloplasty
RAP: Robot-assisted laparoscopic pyeloplasty
FV-UAS: Flexible vacuum-assisted ureteral access sheath
f-URS: Flexible ureteroscopy
IVP: Intravenous pyelography
CT: Computerized tomography
PCNL: Percutaneous nephrolithotomy
VAS: Visual analog scale
IUPA: Infundibulo-ureteropelvic angled
